# Preparation and characterization of nanocurcumin based hybrid virosomes as a drug delivery vehicle with enhanced anticancerous activity and reduced toxicity

**DOI:** 10.1038/s41598-020-79631-1

**Published:** 2021-01-11

**Authors:** Varun Kumar, Ramesh Kumar, V. K. Jain, Suman Nagpal

**Affiliations:** 1 Amity Institute of Advanced Research and Studies (Materials & Devices), Amity University, Noida-201303, UP India; 2 Virology Section, Department of Microbiology, All India Institute of Medical Sciences (AIIMS), New Delhi, India

**Keywords:** Microbiology, Molecular biology, Plant sciences, Medical research, Nanoscience and technology

## Abstract

The present study represents a formulation of nanocurcumin based hybrid virosomes (NC-virosome) to deliver drugs at targeted sites. Curcumin is a bioactive component derived from *Curcuma longa* and well-known for its medicinal property, but it exhibits poor solubility and rapid metabolism, which led to low bioavailability and hence limits its applications. Nanocurcumin was prepared to increase the aqueous solubility and to overcome all the limitations associated with curcumin. Influenza virosomes were prepared by solubilization of the viral membrane with 1,2-distearoyl-*sn*-glycerol-3-phosphocholine (DSPC). During membrane reconstitution, the hydrophilic nanocurcumin was added to the solvent system, followed by overnight dialysis to obtain NC-virosomes. The same was characterized using a transmission electron microscope (TEM) and scanning electron microscope (SEM), MTT assay was used to evaluate it's in vitro-cytotoxicity using MDA-MB231 and Mesenchyme stem cells (MSCs). The results showed NC-virosomes has spherical morphology with size ranging between 60 and 90 nm. It showed 82.6% drug encapsulation efficiency. The viability of MDA-MB231 cells was significantly inhibited by NC-virosome in a concentration-dependent manner at a specific time. The IC50 for nanocurcumin and NC-virosome was 79.49 and 54.23 µg/ml, respectively. The site-specific drug-targeting, high efficacy and non- toxicity of NC-virosomes proves its future potential as drug delivery vehicles.

## Introduction

Virosomes are reconstituted viral envelopes consisting of viral glycoproteins and membrane lipids resembling the original virus, but lacking any genetic material making their internal compartment empty^1^. The efficient drug delivery via virosomes can be ascribed to their nature of mimicking the natural way of infection by any virus, enabling them to specifically bind with target cell surface receptors for their entry inside the cell^2^.

Virosome based delivery methods were originally used in nanomedicince, specially for nanoparticle-mediated drug delivery to overcome the various disadvantages normally faced while using conventional drug delivery systems^3,4^. This mode of delivery is quite versatile and specific due to their binding ability of binding with number of receptros including cytokines and also monoclonal antibodies which are specific for tumor cells (Fab fragments), making them highly specific for targeted tumor cell^5^.

One of the most frequently used virosomes for drug delivery are Influenza virosomes. Their envelope comprises a phospholipid bilayer along with the two viral glycoproteins, namely, hemagglutinin (HA) and neuraminidase (NA) protruding on the surface^6–8^. The primary role of HA is that of receptor-binding and fusion with the host cell’s membrane, followed by receptor-mediated endocytosis; HA can form homotrimers and becomes fusion-competent at acidic pH by changing its conformations, in addition to providing structural stability and homogeneity to formulations of virosome^2,9^. The second glycoprotein present, NA, not only helps in the entry of virus into the targeted cells^9,10^ but also helps in removal of terminal sialic acid from the saccharide chain present on the surface of host cell for comfortable release of newly-formed virus particles during the budding stage^11^. Although virosomes are well known to bind with several synthetic drugs (doxorubicin, methotrexate etc.) by earlier authors for their controlled and targeted delivery^12,13^, but there is no report available in literature for any herbal drug encapsulation into the virosomes.

Anti-proliferative medicinal active compounds with high efficacy and negligible adverse effects on the immune system either de-novo or by purification has emerged as an important branch of immunopharmacology^14^. Curcumin (*Curcuma longa*), as known for centuries, is an extremely potent, nontoxic, anti-inflammatory, antimicrobial, and anti-cancer agent^11^ but it has few drawbacks such as low aqueous solubility and poor bioavailability, thus hindering its clinical applications^15,16^. However, these drawbacks of curcumin were overcome by us in our earlier work by the preparation of nanocurcumin via wet-milling method^17,18^. The bioavailability of nanocurcumin can also be improved by incorporating the prepared curcumin nanoparticles into the influenza virosomes while reconstituting the viral membrane moreover, it can be expected to make the drug delivery at target cells also with much more efficient way.

Therefore, in this manuscript, we prepared the Virosome by the membrane solubilization and reconstitution method and filled it with the herbal drug (nanocurcumin) for site-specific targeted delivery. NC-Virosome were characterized using different analytical tools. The efficacy of free nanocurmin and NC-Virosome were studied on MDA-MB-231 and MSC’s (mesenchyme stem cells) cell line by using 3-(4,5-dimethylthiazol-2-yl)-2,5-diphenyltetrazolium bromide (MTT) assay to identify their anticancerous potential with no toxic effect on primary cells. The efficacy and site-specific drug delivery of nanocurcumin and NC-Virosome were compared and correlated using MTT assay.

## Results

### Characterization of the prepared nanocurcumin

The nanoparticles of curcumin were prepared using wet milling process and characterized by various techniques such as.

#### UV–visible spectroscopy

**Figure 1:**
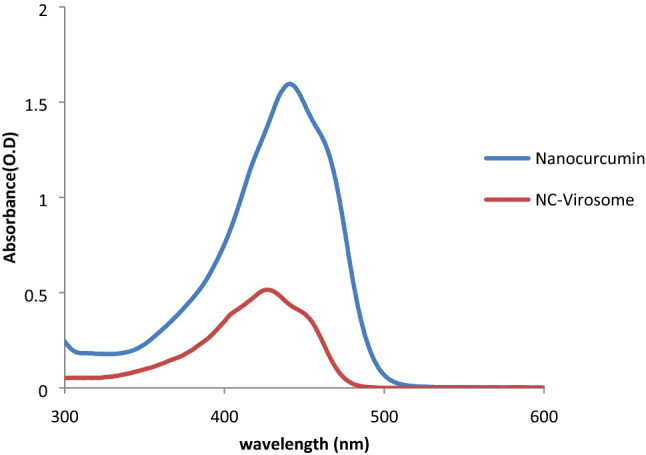
UV–visible spectrum showing a characteristic peak of nanocurcumin at 435 nm and a shifted peak at 419 nm for NC-virosome.

The prepared nanocurcumin sample was subjected to UV–visible spectroscopy analysis. The UV–visible spectrum showed the characteristic peak at 435 nm (Fig. 1).

#### Dynamic light scattering (DLS)

**Figure 2:**
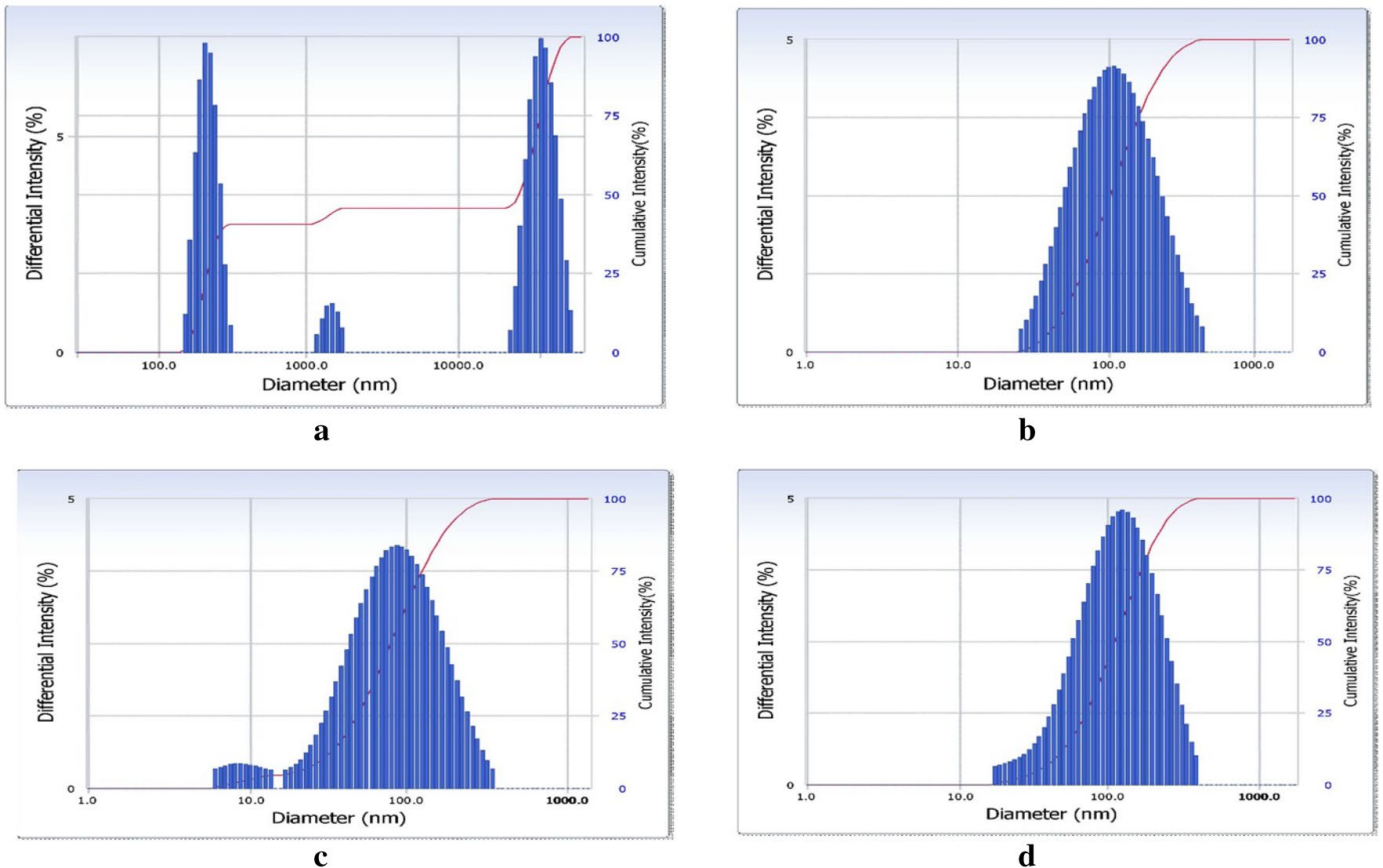
Dynamic light scattering showing intensity distribution graph representing the average hydrodynamic size of (**a**) curcumin; (**b**) nanocurcumin particles; (**c**) empty-virosome; (**d**) NC-virosome.

DLS was used to determine the size distribution profile of nanoparticles in a suspension or solution via a laser or a monochromatic light source. DLS of an aqueous dispersion of nanocurcumin and bulk curcumin showed an average hydrodynamic diameter of 74.9 nm and 2.45 µm, respectively (Fig. 2a,b).

#### Scanning electron microscopy (SEM)

**Figure 3:**
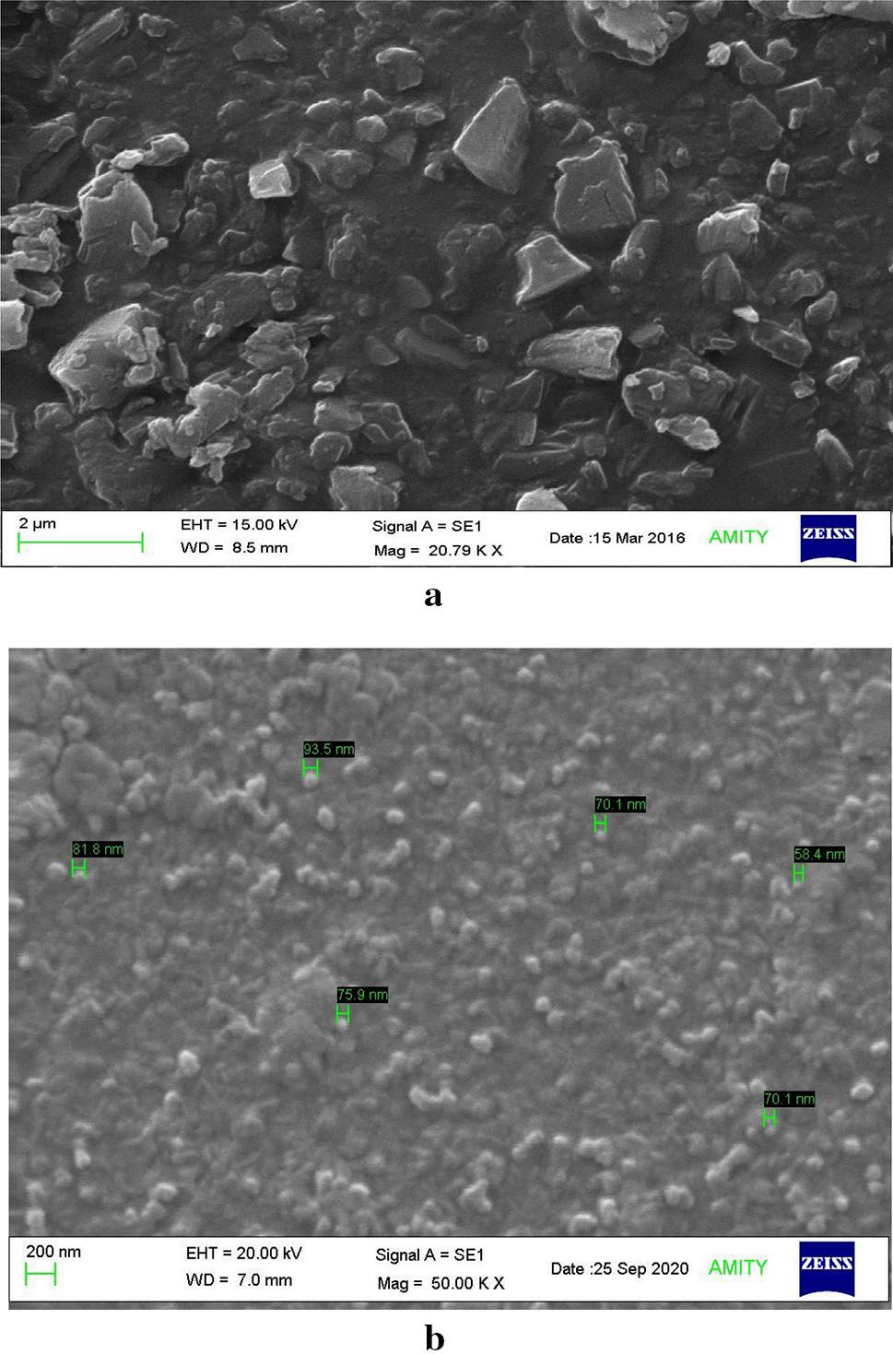
SEM images showing the difference in surface morphological, shape, and size of (**a**) curcumin powder; (**b**) and nanocurcumin particles.

The structural morphology of bulk curcumin powder and its particle size was analyzed using SEM and compared with that of nanocurcumin. The result showed that in the case of curcumin powder, no uniform particle shape was observed and the particles size was ranging from 0.5 to 2 µm in diameter (Fig. 3a), while uniform spherical morphology was observed in the case of nanocurcumin particles with size ranging from 65 to 75 nm, indicating the successful preparation of curcumin nanoparticles (Fig. 3b).

### Characterization of NC-virosomes

#### Bradford’s assay

The total protein concentration of the NC-virosome, as well as that of the whole virus, was determined by Bradford's assay. The equation for a straight line (linear absorbance of BSA): y = mx + c and y = 0.001x + 0.0739 was obtained by substituting the values, where "y" corresponds to absorbance and "x" corresponds to protein concentration. The results showed that purified virosomes (i.e. empty virosome) contained approximately 167 µg/ml protein concentration, which was found to be 52.18% of the initial membrane protein (i.e. 320 µg/ml) in whole influenza virus (Table 1).

**Table 1:** Protein concentration and absorbance of the whole virus and virosome samples.

Sample	Absorbance (595 nm)	Concentration (µg/ml)
Influenza virus	0.393	320
Empty virosome	0.240	167
Virosome + nanocurcumin	0.264	169

#### UV–visible spectroscopy

The prepared NC-virosome as well as nanocurcumin samples were subjected to UV–visible spectroscopy analysis. The results showed a characteristic peak of nanocurcumin at 435 nm, while NC-virosomes showed a well-demarked peak at 419 nm. During the primary analysis, it was observed that the initial peak of nanocurcumin at 435 nm has been shifted to 419 nm after virosomal encapsulation (Fig. 1).

#### Dynamic light scattering (DLS)

DLS was used to determine the size distribution profile of empty-virosome and NC-virosome via laser or a monochromatic light source. DLS of an empty-virosome and NC-virosome showed an average hydrodynamic diameter of 68.6 nm and 91.1 nm, respectively (Fig. 2c,d).

#### Scanning electron microscopy (SEM)

**Figure 4:**
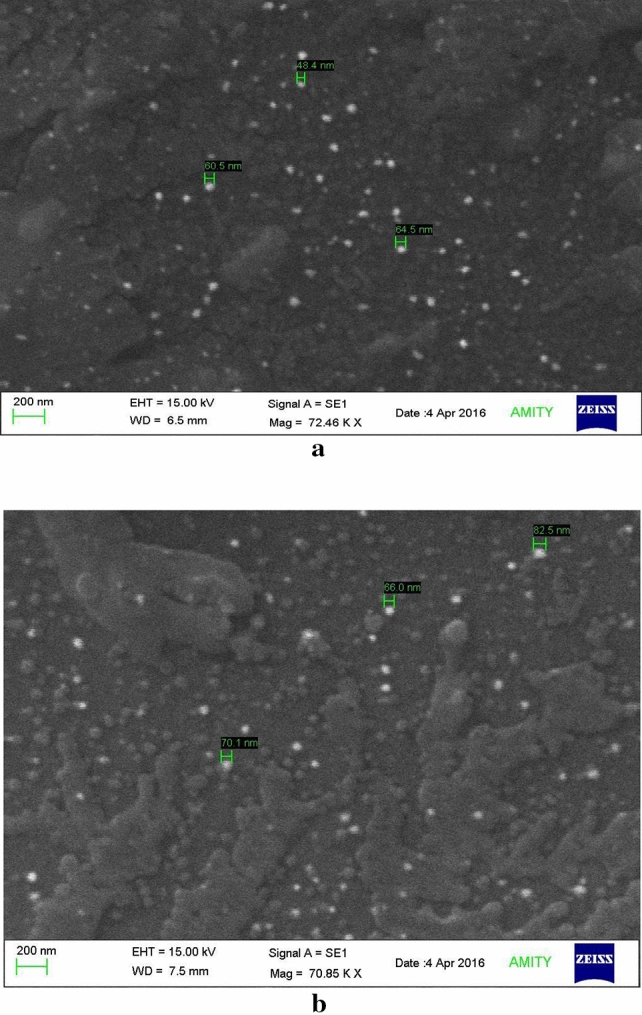
(**a**) SEM image of empty reconstituted virosome-SEM image depicts a reduction in particle size of reconstituted virosome (in white), with size ranging from 40 to 65 nm, due to the loss of membrane proteins and lipids; (**b**) SEM image of virosome with nanocurcumin sample shows circular morphology and size ranging from 60 to 90 nm.

SEM results for the empty reconstituted virosome showed well-dispersed particles with spherical morphology, having particles size ranging from 48-64 nm (Fig. 4a). Similarly, the SEM results for NC-virosomes showed spherical structural morphology, with particle sizes ranging from 60 to 90 nm (Fig. 4b).

#### Transmission electron microscopy (TEM)

**Figure 5:**
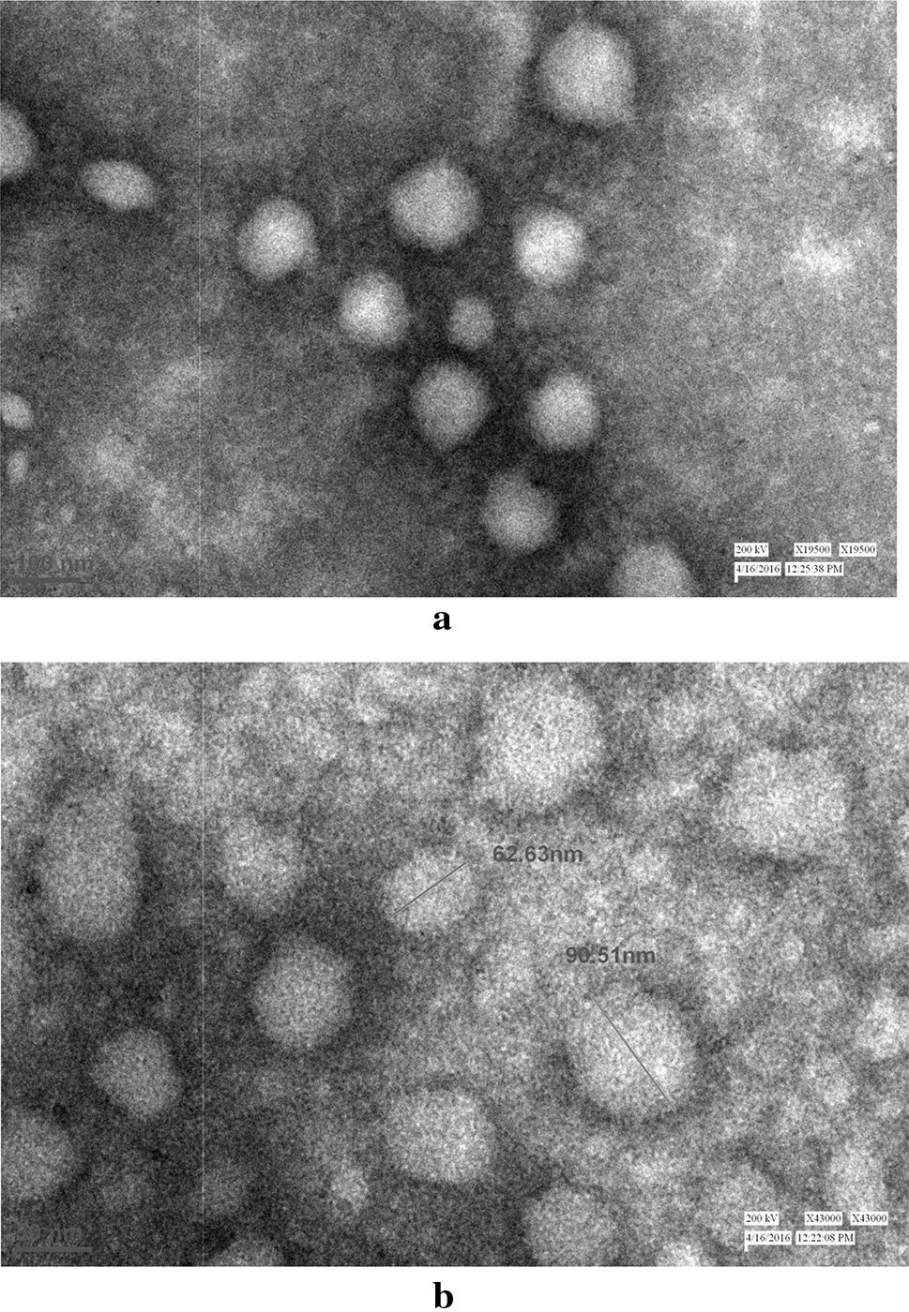
(**a**) TEM image for an empty influenza virosomes-showed the negatively stained sample confirms the formation of empty influenza A/H1N1 virosome particles, hollow from the interior with their reconstituted membranes; (**b**) TEM image of virosome with nanocurcumin sample-showed reconstituted virosome sample (in white) with nanocurcumin.

The TEM analysis also showed a spherical morphology with an average diameter of 40–60 nm for the empty-reconstituted influenza virosome (Fig. 5a). The formation of empty-virosomes, with their hollow interior and a faint, yet visible membrane outline further strengthens this part of the study. The results obtained for the prepared influenza virosome particles strongly asserted the formation of empty virosomes (Fig. 5a). Whereas, TEM results obtained for the NC-virosome showed a uniform spherical morphology with the differentiated outer membrane and a size ranging from 60 to 90 nm in diameter. The internal glowing compartment depicted the nanocurcumin encapsulation into the reconstituted influenza virosome, as nanocurcumin glows under the influence of light (Fig. 5b).

### The encapsulation efficiency (EE) of NC-virosome

The absorbance at different concentrations of nanocurcumin was measured using a UV–visible spectrophotometer at 435 nm. The standard curve of nanocurcumin (10–100 ng/ml) was prepared by plotting optical density against the concentrations and same was used for determining the concentration of nanocurcumin in the supernatant. The results showed that the amount of nanocurcumin encapsulated before membrane reconstitution was higher than the encapsulation efficiency during membrane reconstitution of virosomes (Table 2). The nanocurcumin encapsulation efficiency during membrane and before membrane reconstitution was estimated to be 50.1% (46.24 ng/ml) and 82.6% (76.57 ng/ml), respectively.

**Table 2:** Encapsulation efficiency of the influenza virosome.

Virosomal encapsulation	Mean particles size (nm)	Polydispersity Index (PDI)	Zeta potential	Encapsulation efficiency	
				Before reconstitution	After reconstitution
Nanocurcumin	74.9 ± 3.06	0.512 ± 0.03	− 61.0 ± 0.68	82.6%	50.1%
Empty virosome	85.1 ± 4.65	0.520 ± 0.03	− 56.63 ± 0.06	–	

### In-vitro drug release study of NC-virosome

**Figure 6:**
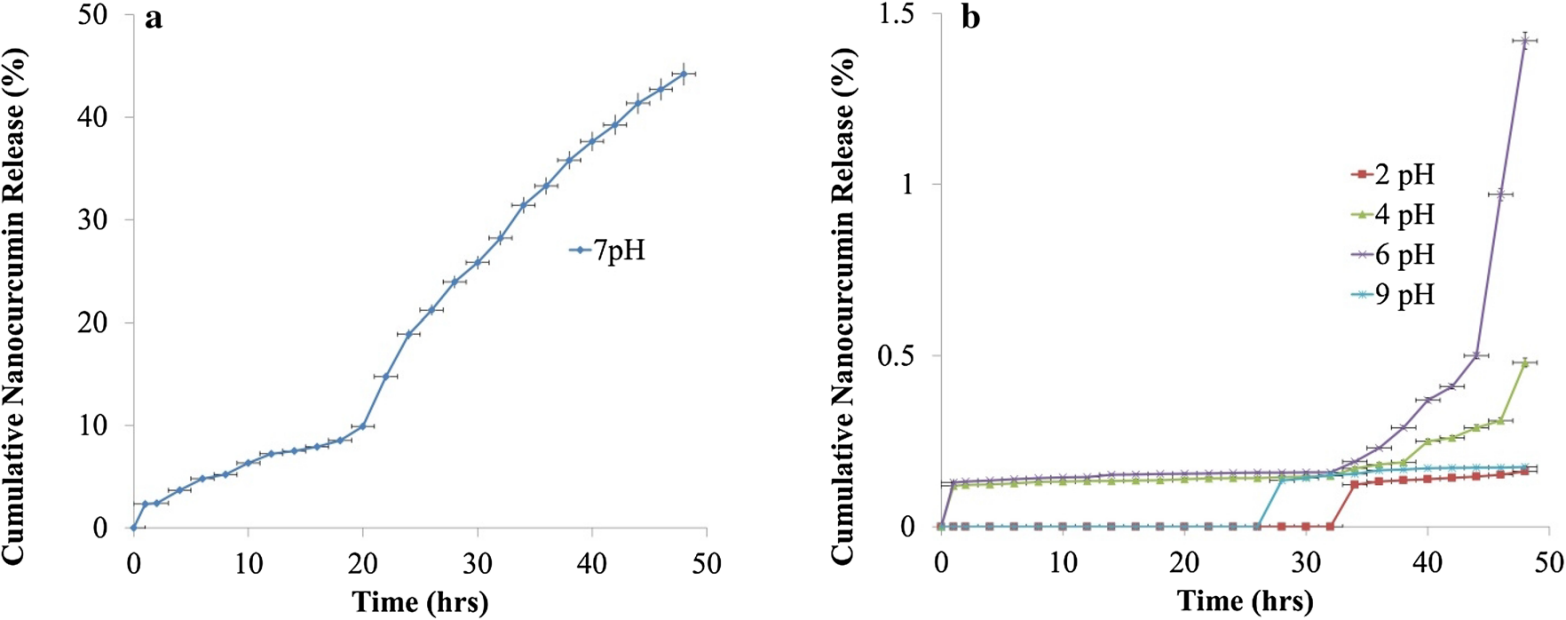
(**a**) Cumulative nanocurcumin release profile from influenza virosome monitored through the dissolution method at pH.7 (**b**) and pH. 2, 4, 6, 7, and 9 at the wavelength of 435 nm. Here, the error bar represents the standard error within the observations. The data were performed in triplicates.

In-vitro drug release study of nanocurcumin has been evaluated over complete pH range, ranging from highly acidic to basic by taking specific intervals at pH 2, 4, 6, 7, and 9. The reported release of the drug was 18.87% and 44.2% from NC-virosome at pH 7.0 after 24 h and 48 h, respectively (Fig. 6a). Whereas, there was a negligible release of drug at pH 2, 4, 6, and 9 even at 48 h (Fig. 6b).

### The anti-proliferation effect of nanocurcumin, empty-virosome, and NC-virosome on MDA-MB231 and MSCs cell lines

**Figure 7:**
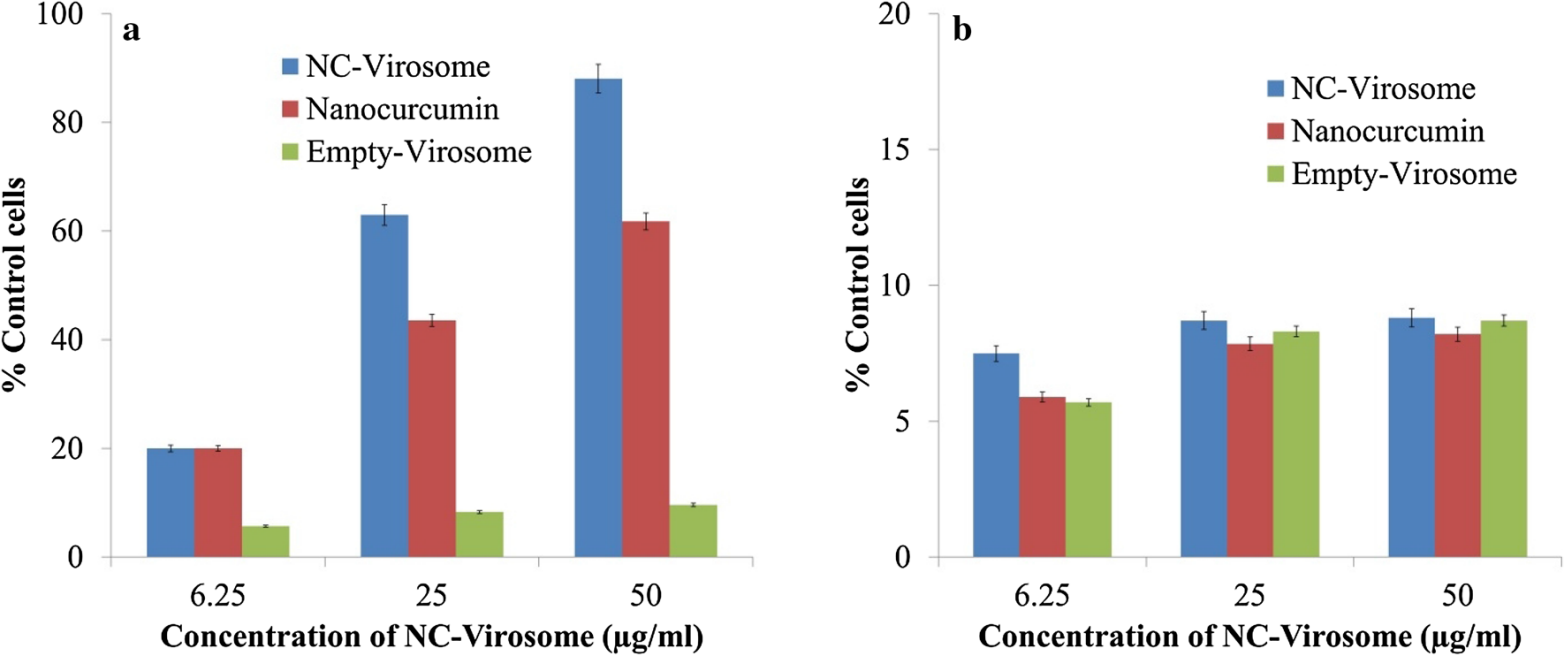
(**a**) The graph showing % cell growth control with the different concentrations of the nanocurcumin, empty-virosome, and NC-virosomes on MDA-MB231 cells at 48 h. (**b**) The graph showing % cell growth control with the different concentrations of the nanocurcumin, empty-virosome, and NC-virosomes on MSCs cells. The statistical analysis was performed through students' t-test, ^#^P ˂ 0.05, ^##^˂ 0.001 compared to control. The error bar represents standard error among the observations. The data were performed in triplicate.

The anti-proliferation effect of nanocurcumin, empty-virosome, and NC-virosome was studied in a dose-dependent manner. The proliferation of MDA-MB231 cells was significantly inhibited by both the nanocurcumin and NC-virosome in a concentration-dependent manner (P < 0.01) at a specific time (Fig. 7a,b) with no significant cytotoxic effect on MSCs (primary cell lines). Whereas, the proliferation rate of MDA-MB231 and MSC’s cells was remained unaffected by the empty-virosome (Fig. 7a,b).

The IC50 value for nanocurcumin as calculated using a linear interpolation method after 48 h on the MDA-MB231 cell line was found to be 79.58 μg/ml while for NC-virosome it was 37.75 μg/ml which is nearly 50% less than that of the nanocurcumin (P < 0.01).

### Cellular uptake of NC-virosome in MDA-MB231 and MSC’s cells

In cells, cellular uptake was analyzed to check the ability of nanocurcumin and NC-virosome to enter the MDA-MB231 and MSC’s cells. The readings were taken at 435 nm by using a UV–visible spectrometer and fluorescence microscope (as nanocurcumin gave green fluorescence due to its chromophore group) after 1 h and 24 h to study drug internalization.

The results showed the maximum absorbance of 0.68 by MDA-MB231cells and 0.16 by MSC’s cells after 24 h of the treatment with 50 μg/ml of NC-virosome formulation at 435 nm, while the concentration of NC-virosome was reached up to 42.7 μg/ml in MDA-MB231 cells and 10.7 μg/ml in MSC’s cells after 24 h of the treatment (Table 3).

**Table 3:** Cellular uptake, absorbance, and fluorescence intensity variations of NC-virosome in different cells.

Cell type		Cell count/unit area	Absorbance Mean ± SD	Concentration (µg/ml)	% Cellular uptake	Integrated density	Difference (treated-control)	CTCF
**Time (h)**								
1	Control	23	NA	NA	NA	407.67	NA	407.67
	MDA-MB231	45	0.129 ± 0.002	8.2	16	33,378.8	32,971	22,550.03
	MSC’s	25	0.019 ± 0.001	˂ 10	˂ 10	21,837.8	21,430.13	2093.13
24	Control	43	NA	NA	NA	7514.667	NA	6751.956
	MDA-MB231	66	0.682 ± 0.015	42.17	84	153,004.8	145,490.8	138,294.9
	MSC’s	25	0.161 ± 0.002	10.7	21.40	123,061	115,547	6338.102

The mean fluorescence intensity per unit area for both the cells was calculated with the aid of a FiJi ImageJ analytical tool and results are listed in Table 3. After 24 h of treatment, fluorescence intensity was estimated and the mean values are 138,294.9 and 6338.1 for MDA-MB231 and MSC’s, respectively (Table 3).

**Figure 8:**
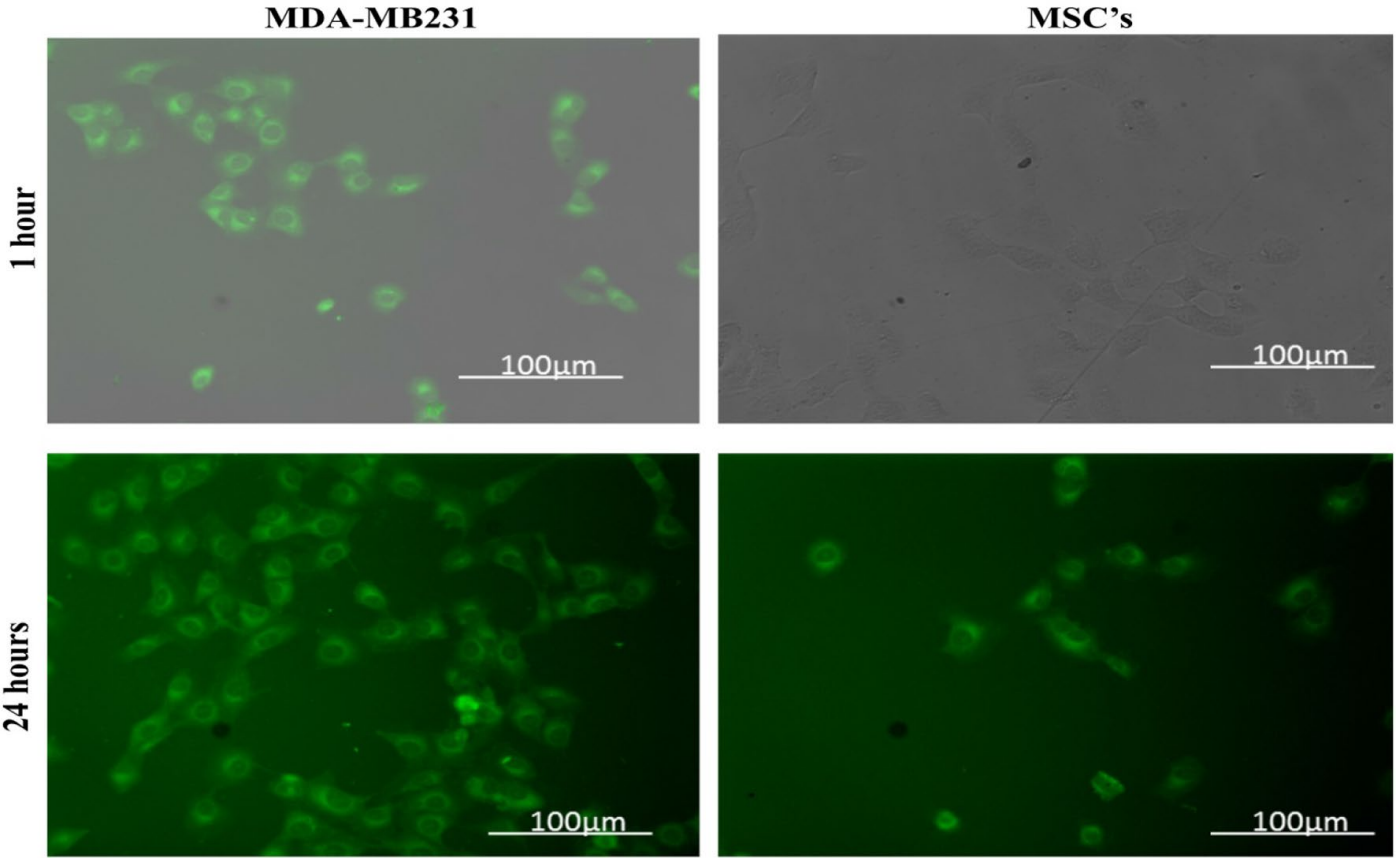
Cellular uptake of NC-virosome in MDA-MB231 and MSC’s cells through a fluorescence microscope (objective lens 60×) at different time points (1 and 24 h).

The results indicated that the nanocurcumin fluorescence could be observed within cells as early as just after 1 h and the intensity was also found to be uniformly distributed throughout the cell. Over the time from 1 to 24 h, the intensity seems to be more localized into cellular compartments (Fig. 8).

## Discussion

The UV–visible spectrum indicated the successful preparation of nanocurcumin by showing a characteristic peak at 435 nm and DLS of the nanocurcumin aqueous dispersion revealed an average hydrodynamic size of 74.9 nm. In the case of NC-virosome, the characteristic peak of nanocurcumin has been shifted to 419 nm from 435 nm. This shift was observed after the influenza virosomal encapsulation. The shift in the peak of the spectrum indicates either modifications in the structure or chemical environment of the nanocurcumin. A shift in UV–visible spectrum of nanocurcumin was also observed after its encapsulation inside the virosome which might be due to some chemical structural rearrangement or could be possible that nanocurcumin reacts with the radicals formed during the membrane solubilization and reconstitution process and as a result, it might rearrange its active chemical group making them more exposed which may lead to the enhanced cytotoxic activity of the compound.

Moreover, lyophilized powder of the nanocurcumin was found to have good physical and chemical stability and could be stored at room temperature for over 6 months without any aggregation. The increased aqueous solubility of nanocurcumin resulted due to their large surface area, which endorses dissolution^19^. Similar results were observed in previous studies where the efficacy, solubility, and bioavailability of nanocurcumin was reported to be enhanced with a reduction in the particle size^20^.

SEM analysis of the bulk curcumin powder showed an uneven shape with large particles nearly 0.5–2 µm in diameter. While after converting into nanocurcumin, particles were found to be well dispersed with spherical morphology and the particle size ranging from 65 to 75 nm; indicating the successful preparation of nanocurcumin.

The data from Bradford’s assay showed the reconstituted NC-virosomes contained approximately 53.56% or 169 µg/ml protein concentration as compared to the whole virus sample. This was observed as the positive indication in the virosome formation as the membrane protein concentration tends to reduce, approximately by 50–70% during membrane reconstitution, since a considerable amount of membrane glycoproteins and lipids were lost during dialysis and ultracentrifugation.

Further results obtained using SEM analysis showed that the empty reconstituted virosome with spherical particles, ranging from 48 to 64 nm. The observed particle size for the empty-virosome is one-third of the whole influenza virus size ranging from 190 to 200 nm as reported by Lamb et al.^10^. The size reduction can be directly correlated with the considerable loss of membrane lipids and glycoproteins, also shown by Bradford's assay. The reconstituted membranes, thus, have varied structural morphologies and reduced size ascribing to a reduction in membrane protein concentrations. While in the case of NC-virosomes, the sample showed a spherical shape, with particle size ranging from 60 to 90 nm. The particle size of the NC-virosome sample was found out to be bigger when compared to the empty reconstituted virosomes, the probable reason being the addition of nanocurcumin particles into the solvent system. With the average particle size of nanocurcumin being 74.9 nm, it may be regarded that the viral membrane could have been reconstituted with some amount of nanocurcumin integrating or conjugating with the viral membrane glycoproteins and lipids, thus, increasing the particle size of the resulting virosomes formed. The TEM analysis of the prepared virosomes, with their hollow interior and a faint, yet visible membrane outline further strengthens this part of the objective. Whereas results obtained for NC-virosome depicted the successful encapsulation of nanocurcumin into virosome.

The nanocurcumin encapsulation efficiency during membrane reconstitution was estimated to be 50.1% (46.24 ng/ml) and before membrane reconstitution, it was 82.6% (76.57 ng/ml), which is much higher than the earlier reported encapsulation for bulk curcumin inside the Diblock Copolymer Micelles^21^ and approximately 50% more efficient in controlled drug release than the previously reported liposome based drug delivery vehicle^4,15^. Therefore nanocurcumin encapsulation before membrane reconstitution was considered as the optimum method for further study.

The in vitro drug release profile of the nanocurcumin showed maximum drug release at pH 7.0 and there is no or negligible drug release from NC-virosome at pH 2, 4, 6, and 9. Studies conducted over the last few years have demonstrated that the intracellular pH (pHi) of the tumor cell is maintained within a pH range of 7.0–7.2, while the extracellular pH (pHe) is acidic^22^. The prepared NC-virosome only release drug inside the tumor cell and simultaneously preventing its leakage in the extracellular environment, hence making virosomes a good choice for delivering biological as well as a synthetic drug by manifesting pH specific release of the drugs to targeted sites along with no side effects on normal tissues.

**Figure 9:**
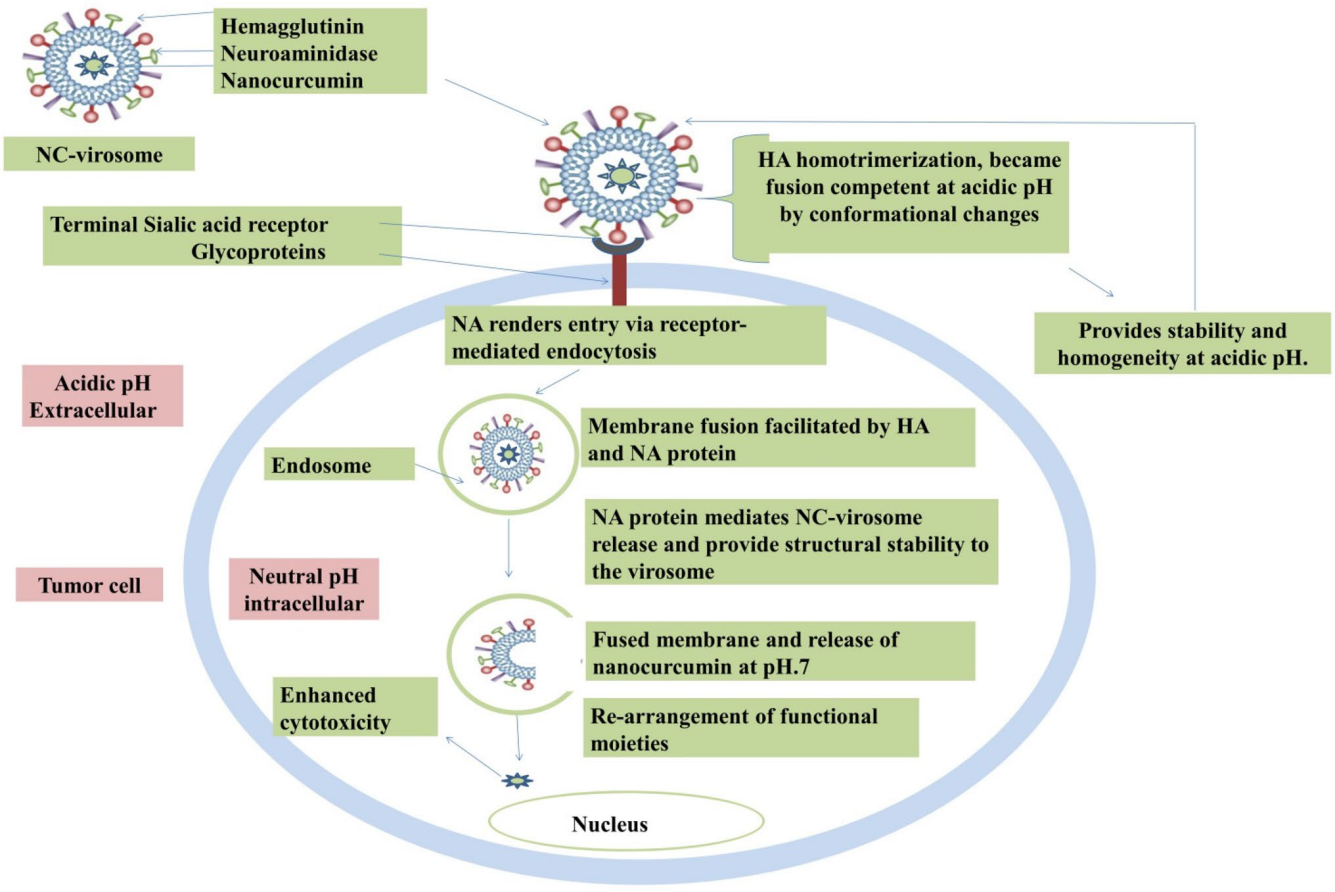
The mechanism of NC-virosome entry and nanocurcumin release inside the tumor cell.

The release mechanism involves the binding of virosomes through hemagglutinin (HA) to the cell membrane receptors that are glycoproteins/glycolipid with terminal sialic acid. The virosomes entry occurs through receptor-mediated endocytosis. Virosomes are trapped inside the endosomes; the environment inside the endosomes renders the membrane fusion activity between virosomes and endosomes. The fusion activity is facilitated by the viral membrane glycoprotein hemagglutinin (HA). The resulted membrane-fusion activity in the endosome releases the virosome from its lipid envelope and offers access for the encapsulated drugs to the cytoplasm of the cell^23^ (Fig. 9).

Moreover, in our study shielding effect of the lipid bilayer as usually observed in liposomes mediated drug delivery method^24,25^ was found to be overcome by nanocurcumin encapsulation into the virosomes which showed a synergic hybrid combination leading to sustained and prolong drug release pattern to the targeted sites without affecting the physiology of normal cells.

The significant difference in IC50 of NC-virosomes, proved them to be 32% more effective compared to the nanocurcumin alone (P < 0.01). The enhanced cytotoxicity and targeted delivery of nanocurcumin to MDA-MB231 cells by NC-virosome resulting from the activity of two viral glycoproteins, namely, hemagglutinin (HA) and neuraminidase (NA) protruding on the surface. The primary role of HA is that of receptor-binding and fusion with the host cell's membrane, followed by receptor-mediated endocytosis of nanocurcumin to the MDA-MB231 cells.

The cellular uptake data at 1 h and 24 h, suggests the maximum release of nanocurcumin in both MDA-MB231 and MSCs cells from hybrid virosome occurred at 24 h of the drug treatment. This can be seen that the MDA-MB231 cells showed a significantly 73.7% higher uptake of NC-virosome as compared to MSC’s cells at 24 h of the treatment (P < 0.002). The observations also showed that the uptake increased with increasing concentration of NC-virosome treatment in both the cells with different incubation times. The high absorbance and fluorescence intensity was observed in the tumor cell lines (MDA-MB231). One of the main reasons for such higher fluorescence intensity and absorbance could have resulted from the increased cellular uptake. To verify this, the fluorescence intensity was determined to the same level of NC-virosome uptake and compared the fluorescence intensity per unit uptake. This can be concluded from the observations that the fluorescence intensity is 94.5% more intense in the MDA-MB231 cells than that in MSC’S cells. The change in intensity variations clearly showed that NC-virosome encounters different internal environments in MDA-MB231 cells and interacts differently.

The obtained results favored our observations that NC-virosome exhibits higher absorbance in MDA-MB231cells. A trial to trace the cellular localization of nanocurcumin proved that over the time (1–24 h), intensity seems to be more localized into cellular compartments. This makes us hypothesize that a treatment time of 24 h is optimum for maximum cellular uptake of NC-virosomes in tumor cells. Hence, the site-specific, drug-targeting potential, and high efficiency of NC-virosomes proved as the future potential for targeted drug vehicles without any toxic effect on normal cells.

## Conclusion

We prepared a novel Influenza A/H1N1 herbal hybrid virosome using DSPC, a short-chain dialyzable phospholipid and characterized the same with a blend of characterization tools. The novel herbal hybrid combination proved to be more efficient in controlled drug release. The viability of MDA-MB231 cells was significantly inhibited by nanocurcumin encapsulated influenza A/H1N1 virosome in a concentration-dependent manner in defined times. The significant difference in IC50 of NC-virosome proved them a more effective targeted delivery vehicle as compared to other vehicles available to encapsulate nanocurcumin. The high drug-targeting potential and efficiency demonstrates the significant role of the anticancer properties of nanocurcumin encapsulated influenza A/H1N1 virosome. The ability of influenza virosomes to fuse with the host cell sialic acid receptors can further be exploited to deliver several biopharmaceutically active compounds via receptor-mediated endocytosis. This study is the first step towards producing virosomes encapsulating a herbal drug, thereby significantly increasing their application as therapeutic agents for many diseases, including cancer. Virosomal encapsulation of nanocurcumin not only overcomes the poor solubility issues but also provide controlled and targeted drug release along with protection from the shielding effect exerted by the lipid bilayer membrane. The findings in this study can form the basis for further research and could be used to evaluate the capability of the produced virosomes to function as the agents for cell-specific targeted drug delivery.

## Materials and methodology

### Materials

Curcumin [(1E,6E)-1,7-Bis(4-hydroxy-3-methoxyphenyl)-1,6-heptadiene-3,5-dione] and DSPC (1,2-Distearoyl-*sn*-glycerol-3-phosphocholine were purchased from Sigma Chemicals, India. Dimethyl sulphoxide, Trypan blue, and methylthiazoly-diphenyl-tetrazolium bromide (MTT) were procured from Himedia, India. Dialysis membrane, Dulbecco's Modified Eagle Medium (DMEM), Antibiotics-antimycotics mixture, and fetal bovine serum (FBS) were purchased from GibCo, India.

### Viral strain and mammalian cell lines

The virosomes were prepared from influenza A/H1N1(inactive) strain provided generously by All India Institute of Medical Science, New Delhi, India. MDA-MB-231 and MSC’s (mesenchyme stem cells/Primary cell) cell lines were procured from NCCS, Pune.

### Instruments and equipment

Scanning Electron Microscope (EVO-18, Carl Zeiss, Germany) and FEI Tecnai Transmission Electron Microscope, G2-series, USA were used to study the surface morphology of curcumin nanoparticle and prepared virosome. Dynamic Light Scattering (DLS, Malvern Zetasizer S90 series) to carry the particle size analysis of the prepared nanocurcumin and virosome. An ultrasonic device, ultrasonic cleaner TPC-25 from RoopTelesonic Pvt Ltd., India, used in the preparation of curcumin nanoparticles. Rotavapor (R-210) equipped with a heating bath (B-491) and vacuum pump (V-700), used to concentrate the nanocurcumin sample and to further breakdown the particle size. UV–visible spectrophotometer (Spectro UV–Vis Dual beam and Auto Cell UVS-2700, Labomed, INC, Germany).Co_2_ incubator (MCO-5M MCO Multi-gas incubator) were used for cell culture. The ImageJ (FiJi) software was used to calculate mean fluorescent intensity of the all treated groups.

### Methodology

#### Preparation of nanocurcumin

The nanocurcumin was prepared by using wet milling process (patented process)^26^. Curcumin (100 mg) was dissolved in dimethyl sulfoxide (20 ml). 1 ml of this solution was added into boiling water (50 ml) dropwise with a flow rate of 0.2 ml/min under ultrasonic conditions with an ultrasonic power of 100 W and a frequency of 30 kHz. After addition, the contents were sonicated for 10 min and then stirred at room temperature for about 20 min. The solution was concentrated under reduced pressure at 50ºC and finally freeze-dried to obtain a yellowish-orange powder^26^.

#### Characterization of nanocurcumin

The mean particle size of the nanocurcumin was observed by Dynamic light scattering (DLS). In brief, the sample was prepared by taking 1 mg of the lyophilized nanocurcumin powder in 10 ml (autoclaved) distilled water and used in characterization studies. Scanning Electron Microscopy (SEM) was performed by spreading the nanoparticle dispersion over a carbon tape and drying it under a nitrogen stream. The sample was then coated in a sputter with a gold layer in a vacuum condition^26^.

#### Preparation of NC-virosome

NC-virosomes were prepared using the Membrane Solubilization and Reconstitution Method with modifications^27–29^.

##### Inactivation of virus

Inactivation of the Influenza A/H1N1 sample was performed by adding 0.02% formaldehyde in a 1:2 ratio and the sample was stored at − 20 °C for three days for complete viral inactivation. 500 µl of the inactive viral sample was transferred aseptically in three autoclaved eppendorf (A, B & C) and ultracentrifuge at 100,000 × *g* for 1 h at 4 °C. The supernatant obtained was discarded (in 0.02% formaldehyde) and the pellet was dissolved in Hanks Buffered Saline solution (HBBS). Eppendorf's A was stored at − 20 °C, to be used as the control sample during characterization.

##### Viral membrane solubilization

The remaining two eppendorf (B & C) were subjected to membrane solubilization, 370 µl of DSPC (1,2-Distearoyl-*sn*-glycerol-3-phosphocholine) was added into each eppendorf and the suspension was subjected to ice incubation for 30 min. The pellet was collected once centrifugation is done at 100,000 × *g* for 1 h and resuspended in HBSS buffer.

##### Encapsulation of nanocurcumin

The crude virosomes sample in eppendorf B marked as blank, virosomes without any drug and in another Eppendorf C 200 µl, nanocurcumin was added and marked as NC-virosome. Both the preparations (B&C) were separately applied to a discontinuous sucrose gradient (10%/50% w/v sucrose in HBSS). The preparations were prepared in the following order: 130 µl of 50% sucrose + 170 µl of 10% sucrose + 200 µl virosome sample containing nanocurcumin; 130 µl of 50% sucrose + 170 µl of 10% sucrose + 200 µl virosome sample not containing nanocurcumin. The samples were then subjected to ultracentrifugation at 100,000 × *g* for 1.5 h with a temperature of 4 °C, the intermediate layer obtained after discontinuous centrifugation was collected and stored at 4 °C followed by the dialysis in PBS buffer (pH 7.0) for 72 h with subsequent removal and addition of buffer at regular interval of 8 h.

The encapsulation of nanocurcumin within the virosome was performed in two different ways: before membrane reconstitution and during membrane reconstitution. Before membrane reconstitution, nanocurcumin (0.1 ml) was added just before the discontinuous sucrose gradient centrifugation whereas, during membrane reconstitution nanocurcumin (0.1 ml) was added at the time of dialysis.

#### Characterization of virosomes and NC-virosomes

##### Bradford’s assay

The method of protein estimation is based on the binding of Coomassie Brilliant Blue G-250 dye to the protein present in the sample. This bound form of the dye has the maximum absorption spectrum at 595 nm. This increase in the absorbance at 595 nm is directly proportional to the concentration of protein present in the sample (Bradford, 1976). Therefore, this assay was used to analyze the protein content of an intact influenza virus sample (used as control), the prepared empty influenza virosomes and NC-virosomes, and the readings obtained were marked against the standard protein curve prepared by serial dilution of Bovine Serum Albumin (BSA).

##### UV–visible spectroscopy

The prepared NC-virosome sample was subjected to UV–visible spectroscopy analysis to confirm nanocurcumin encapsulation into the virosomes. The UV–visible spectrum of nanocurcumin and NC-virosome were recorded and compared.

##### Dynamic light scattering (DLS)

The prepared empty-virosome and NC-virosome were subjected to DLS for the measurement of an average hydrodynamic size using a monochromatic light source. The DLS results for the prepared empty-virosome and NC-virosome were compared and correlated.

##### Scanning electron microscopy

The prepared samples of the reconstituted empty-virosome and reconstituted NC-virosome were subjected to Scanning Electron Microscopy to determine their structure, surface topology, and size. The results for all samples were compared and correlated.

##### Transmission electron microscopy

The transmission electron microscopy was performed to obtain topographical, morphological, compositional, and crystalline information of the whole influenza virus (as control), reconstituted virosome, and reconstituted NC-virosome. The results for all samples were recorded and compared.

#### The encapsulation efficiency of NC-virosome

The encapsulation efficiency of the NC-virosome was evaluated before (during sucrose gradient centrifugation) and during the membrane reconstitution process through centrifugation method^30^. The centrifugation was done with 1.5 ml of herbal hybrid virosome (with nanocurcumin) for 90 min at 14,000 rpm. The supernatant was taken aseptically to remove untrapped nanocurcumin and their absorbance was checked at 435 nm. The entrapment efficiency of virosome was calculated using:

$${\mathrm{Encapsulation\;efficiency}}=\frac{{\mathrm{drug\;total}}-{\mathrm{drug\;supernatant}}}{\mathrm{drug total}}$$

where drug total is the total amount of nanocurcumin added initially and drug supernatant is the amount of encapsulated nanocurcumin in NC-virosomes^31^.

#### In-vitro drug release study of NC-virosome

The in-vitro drug release study of NC-virosome was monitored through the dissolution method at pH 2, 4, 6, 7, and 9^1,32^. The stock solution of nanocurcumin (1 mg/ml) was prepared in PBS buffer (pH.7) and subsequently diluted to obtain different concentrations ranging from 10 to 100 ng/ml in PBS buffer (pH.7). The standard graph for nanocurcumin was plotted by taking absorbance of various concentrations (10–100 ng/ml) at 435 nm. The pretreatment of the dialysis membrane was done by immersing them in 50 ml of PBS buffer at different pH (2.0–7.0), for at least 25–30 min before the experiment. The dialysis membrane containing 0.5 ml of NC-virosomes was placed in PBS buffer of pH 2, 4, 6, 7, and 9 individually at 37° ± 1 °C with constant stirring at 400 rpm to check the drug release. 1 ml of sample aliquot was withdrawn at regular intervals i.e. 0.25, 0.5, 1–48 h, and absorbance was taken at 435 nm for the evaluation of nanocurcumin release from NC-virosome. The concentration of nanocurcumin in the sample aliquot was quantified by plotting absorbance in the standard graph of nanocurcumin to draw the drug release profile from NC-virosome at different pH (2.0–7.0).

#### Determination of anticancerous efficiency of nanocurcumin, empty-virosome and NC-virosome

##### Maintenance of MDA-MB231 cell line

The MDA-MB231 and MSC's cells were maintained in a DMEM medium containing 10% FBS, 2 mM glutamine, antibiotics, and antimycotic mix under the controlled condition (37 °C, 5% CO_2_, 95% air). Once the cells reached 2.8 × 10^6^ density, were passaged to obtain 5 × 10^3^ number of cells in each well. The cells were treated with 6.25, 25, and 50 μg/ml concentrations of nanocurcumin, empty-virosome, and NC-virosome for 48 h at 37 °C, 5% CO_2_, 95% air in a CO_2_ incubator.

##### Cell viability assay and determination of IC50 value

The anticancerous efficiency of nanocurcumin, empty-virosome, and NC-virosome was determined through cell viability assay (MTT Assay). Briefly, 5 × 10^3^cells/well were inoculated in 96 well plates and treated with 6.25, 25, and 50 μg/ml of nanocurcumin, empty-virosome, and NC-virosome individually in triplicate. After incubation at 37 °C, 5% CO_2_, 95% air in a CO_2_ incubator for 48 h, cells were washed twice with phosphate-buffered saline (PBS) and 100 µl MTT (0.5 mg/ml PBS) was added to each well and again incubated for 4 h at 37 °C. The formazan crystals that formed were dissolved by the addition of dimethyl sulfoxide (100 μl/well) and absorbance was taken by using an ELISA reader (Ebra) at 540 nm. To nullify the test error level, MTT stain was added to some wells with 100% viable cells (control) and without cells (blank). Cell viability was determined by making an average of blank wells containing MTT solution but not MDA-MB231 cells. The absorbance of control wells was measured and ultimately subtracted from the absorbance of blank wells to validate the efficacy of the drug. The cell viability has been calculated through the following formula^33^

$$Cell\;viability={\frac{mean\;absorbance\;of\;treated\;well}{mean\;absorbance\;of\;control\;well}} \times 100\%$$

The IC50 value for NC-virosome, nanocurcumin, and empty virosome in MDA-MB-231 cells was determined by the linear interpolation method as described below^34^

$$IC50=\left[{\frac{50-A}{{\mathrm{B}}-{\mathrm{A}}}}\right]\times \left({\mathrm{D}}-{\mathrm{C}}\right)+{\mathrm{C}}$$

where, A is the point in the curve, demonstrated as percent inhibition which is less than 50%; B is first point in the curve, demonstrated as percentage inhibition which is greater than 50%; C is the concentration of drug that shows A% inhibition and D is the concentration of drug that shows B% inhibition.

#### Cellular uptake of NC-virosome in MDA-MB231 and MSC’s cells

Intracellular uptake of NC-virosome in both MDA-MB231 and MSCs cells was monitored by using a fluorescence microscope. The 5 × 10^4^ cells were seeded in six-well plates with coverslip coated at the bottom of the plates. The cells were incubated in a CO_2_ incubator for 24 h. The culture medium was replaced with a fresh medium containing 50 μg/ml of NC-virosome and incubated in a CO_2_ incubator for 24 h. To estimate the optimum drug internalization through fluorescence intensity, cells were observed under a fluorescence microscope (Zeiss) at the time interval of 1 h and 24 h. To calculate the corrected total cell fluorescence (CTCF) for each treatment, the following formula was used:

$$CTCF = integrated\;density-\left( {area\;of\;selection \times mean\;fluorescence\;of\;background\;readings} \right)$$

For the quantification of cellular uptake standard graph of NC-virosome formulation was prepared by plotting absorbance vs concentrations (5–50 μg/ml) and used to estimate cellular uptake of nanocurcumin. NC-virosome treated cells were spun down (1000 rpm) for 5 min in Beckman centrifuged and washed twice with PBS (pH.7, cold), the pellet was dried and suspended in 1 ml DMSO. The suspension was sonicated for about 20 min or till nanocurcumin is completely extracted in the DMSO fraction. The cell lysate was centrifuged for 10 min at 10,000 rpm, the absorbance of supernatant containing DMSO-nanocurcumin was measured at 435 nm, and the extinction coefficient at 48,000 M/cm. The treatments were performed in triplicate and estimated uptake values for each treatment are represented in Table 3. The control (without drug) and vehicle control (PBS) were kept for each treatment^35^. The Mean fluorescence intensity per unit area for both the cells was calculated with the aid of FiJi ImageJ analytical software. ImageJ is an open-source java-based image processing software designed for scientific multidimensional images. ImageJ is highly extensible, with various plugins and scripts for performing a wide range of scientific activities, including calculation of fluorescence intensity, particle number, size etc.^36^.

A trial has also been done to trace the cellular localization of nanocurcumin as a function of time in MDA-MB231cells. Due to the unavailability of live cell imaging techniques, this could be carried out only by incubating the cells with NC-virosome for two different time periods (1 h and 24 h), and after incubation, fluorescence images were taken. The mean fluorescence intensity/area of MDA-MB231 cells at different incubation times was monitored (Supplementary Information 1).

### Statistical analysis

All the experiments were performed in triplicate and shown values are the mean of the observed triplicate value, whereas the Statistical significance of differences throughout this study was calculated using a Student’s t-test.

### Ethics declarations

No humans and animals were used for the studies.

## Supplementary Information

Supplementary Information 1.

Supplementary Information 2.

Supplementary Information 3.

Supplementary Information 4.

## Data Availability

All data generated or analyzed during this study are included in this published article.
