# Supplementary material for: Preparation and characterization of nanocurcumin based hybrid virosomes as a drug delivery vehicle with enhanced anticancerous activity and reduced toxicity
Source: Sci Rep. 2021 Jan 11;11:368. doi: 10.1038/s41598-020-79631-1 (PMC7801424; doi:10.1038/s41598-020-79631-1)
Supplement: Supplementary file 4 — Supplementary Information 4. [file 41598_2020_79631_MOESM4_ESM.docx]

**Nanocurcumin Entrapment UV-VIS Photometric data**

Standard nanocurcumin photometric absorbance at different concentrations

| **Concentration(ng/ml)** | **Absorbance(nm)** |
| --- | --- |
| 5 | 0.038 |
| 10 | 0.077 |
| 20 | 0.18 |
| 30 | 0.228 |
| 40 | 0.315 |
| 50 | 0.379 |
| 60 | 0.44 |
| 70 | 0.521 |
| 80 | 0.61 |

Photometric absorbance of entrapped nanocurcumin inside the virosome.

| **Nanocurcumin entrapmemt into virosome** | |
| --- | --- |
| Absorbance | 0.609 |
| Concentration | 76.57 ng/ml |

Nanocurcumin standard curve representing absorbance at different concentrations
